# Education and Literacy in the Digital Divide: Cross-National Comparison of East Asia and the USA

**DOI:** 10.1093/geroni/igaa057.1008

**Published:** 2020-12-16

**Authors:** Taka Yamashita, Giyeon Kim, Chih-ling Liou, Takatoshi Ando, Darren Liu, Anthony Bardo

**Affiliations:** 1 University of Maryland, Baltimore, Maryland, United States; 2 Chung-Ang University, Seoul, Republic of Korea; 3 Kent State University at Stark, North Canton, Ohio, United States; 4 Yokohama National University, Yokohama, Japan; 5 Des Moines University, West Des Moines, Iowa, United States; 6 University of Kentucky, Lexington, Kentucky, United States

## Abstract

The disproportionate access to, and utilization of, modern information, communication and computer technologies in later life (or Digital divide) is widely recognized as a major problem in economically developed nations. Extant research has established that the digital divide is largely contingent on advanced age and socioeconomic status (e.g., educational attainment). Yet, most studies have exclusively focused on Western populations. Thus, the objective of this study was to examine patterns in the digital divide by educational attainment, and identify the role that basic skills (i.e., literacy) play in shaping cross-national differences. Internationally representative data of adults aged 45 years and older from Japan, South Korea, and the US were obtained from the Program for International Assessment of Adult Competencies (PIAAC). The PIAAC data include information on everyday digital technology use (e.g., computer device, email, online information and transactions), sociodemographic characteristics, and systematic literacy assessments (score 1-500). Based on results from weighted binary logistic regression models that used the moderator function, the effects of education on email use were significantly weaker (odds-ratio = 0.78, p < 0.05) in Japan vs. US, but equivalent in Japan vs. South Korea (p > 0.05). Additionally, education (odds-ratio = 1.22, p < 0.05) and literacy (odds-ratio = 1.01, p < 0.05) were consistent predictors a digital divide, and the effects of literacy were similar across all three nations (p > 0.05). Theoretical explanations of findings and other results are discussed in cultural contexts.

